# Galantamine-Induced Third-Degree Heart Block

**DOI:** 10.7759/cureus.55757

**Published:** 2024-03-07

**Authors:** Husam Katib, Amna Shah, Hamza Yousaf

**Affiliations:** 1 Internal Medicine, University at Buffalo Jacobs School of Medicine and Biomedical Sciences, Buffalo, USA

**Keywords:** third-degree heart block, alzheimer’s disease, pacemaker, acetylcholinesterase inhibitors, galantamine

## Abstract

Galantamine is commonly used to manage symptoms of Alzheimer’s disease and other cognitive disorders. While it is generally well-tolerated, cardiovascular side effects are rare but can be serious. We report the case of a patient who developed a third-degree heart block after initiating galantamine therapy. This case highlights the importance of monitoring patients for cardiac adverse effects when using galantamine and the need for prompt intervention when such effects occur.

## Introduction

Cholinesterase inhibitors, such as donepezil, rivastigmine, and galantamine, are widely prescribed to improve cognitive function in patients with Alzheimer’s disease and related conditions [1]. While these drugs primarily target the brain, it is important to note that the heart also contains significant amounts of cholinesterases. Inhibiting these enzymes may have adverse effects on cardiac function. These medications are known to increase the availability of acetylcholine in the synaptic cleft, improving cognitive function in some patients. They are generally considered safe, with common side effects being gastrointestinal in nature. However, cardiac side effects, such as bradycardia, syncope, and third-degree heart block, are exceedingly rare [2]. We present a case of cholinesterase inhibitors-induced third-degree heart block, emphasizing the need for vigilance in monitoring patients undergoing this treatment.

## Case presentation

**Figure 1:**
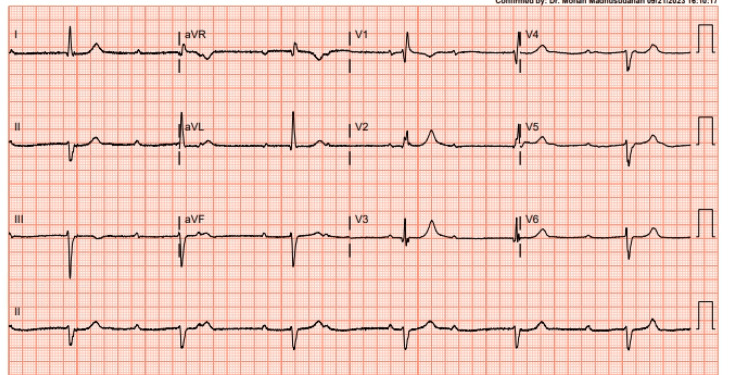
Electrocardiogram showing complete heart block.

A 72-year-old woman with a medical background notable for Alzheimer’s dementia and dyslipidemia was hospitalized for dizziness and difficulty breathing. She had a history of mild aortic valve insufficiency but no reported chest pain or palpitation. She also denied having seizures or syncope. Upon admission, an electrocardiogram revealed a third-degree atrioventricular block and left anterior fascicular block with a ventricular escape rhythm at approximately 40 beats/minute and a relatively narrow QRS with a right bundle branch block morphology and left axis deviation (Figure 1).

Six months before the presentation, she had begun treatment with galantamine and donepezil for her dementia. However, she had ceased taking galantamine for several months before this episode and had resumed it three days before presenting to the emergency department. She had not previously used atrioventricular blockers. Her sole other medication was rosuvastatin. Electrolytes, troponin, brain natriuretic peptide, thyroid function, vitamin B12, folate, and syphilis serology were normal or negative. An echocardiogram demonstrated an ejection fraction of 55-60% with no evidence of wall motion abnormalities or new valvular issues. Orthostatics was negative. It was determined that her complete heart block was likely induced by galantamine. Galantamine was stopped, leading to a gradual rise in heart rate. Temporary bedside transvenous pacing was tried, followed by a permanent dual-chamber pacemaker placement. Galantamine was resumed at discharge after a discussion of the risks and benefits, concluding that the benefits outweighed the risks in her case.

## Discussion

Cholinesterase inhibitors are used to enhance cholinergic function in the central nervous system. Our patient’s presentation of third-degree heart block shortly after cholinesterase inhibitor initiation is consistent with drug-induced adverse effects. The mechanism by which cholinesterase inhibitors may induce heart block is not well understood but may involve its cholinergic effects on cardiac conduction. Elevated levels of acetylcholine can enhance the activity of the parasympathetic nervous system in the sinoatrial node, which leads to a decrease in the sinus rhythm and affects different cardiovascular conduction systems in a supportive manner [3].

In previous case reports, similar cardiovascular events have occurred with cholinesterase inhibitors, in general, and galantamine use, in particular, prompting the need for caution when prescribing this medication, especially in patients with pre-existing cardiac conditions or those taking concomitant medications that affect cardiac conduction.

Patients who are prescribed these medications tend to be older and susceptible to age-related changes that can increase their risk of orthostasis and syncope. These changes include impaired thirst mechanisms, abnormal baroreceptor and autonomic function, and myocardial diastolic dysfunction. Additionally, they may already have underlying cardiovascular disease, which could worsen any predisposition to bradycardia, or there could be interactions with other concurrent medications. Consequently, there are legitimate concerns regarding the potential adverse effects associated with the use of these drugs.

Dunn et al., in their investigation of 1,762 patients with Alzheimer’s disease receiving donepezil, identified nausea, diarrhea, malaise, dizziness, and insomnia as prevalent side effects. Importantly, no instances of cardiac rhythm disturbances were reported [4,5]. Bordier et al. examined 16 patients diagnosed with Alzheimer’s disease who exhibited syncope. Among these cases, an atrioventricular block was found in two out of the 16 individuals [5,6].

## Conclusions

Galantamine-induced third-degree heart block is a rare but potentially serious adverse effect. Clinicians should be aware of the possibility of cardiac side effects when prescribing galantamine, especially in elderly patients with comorbidities. Close monitoring of patients during the early stages of therapy is crucial, and prompt discontinuation of the drug is warranted if cardiac abnormalities are detected. In cases of severe conduction disturbances, as, in this instance, pacemaker implantation may be necessary. Further research is needed to elucidate the mechanisms underlying galantamine-induced cardiac adverse effects and to develop strategies for their prevention and management.
